# Are informal family caregivers stigmatized differently based on their gender or employment status?: a German study on public stigma towards informal long-term caregivers of older individuals

**DOI:** 10.1186/s12889-021-11955-7

**Published:** 2021-10-16

**Authors:** Larissa Zwar, Matthias C. Angermeyer, Herbert Matschinger, Steffi G. Riedel-Heller, Hans-Helmut König, André Hajek

**Affiliations:** 1 Department of Health Economics and Health Services Research, University Medical Center Hamburg-Eppendorf, 20246 Hamburg, Germany; 2 Center for Public Mental Health, Gösing am Wagram, Austria; 3 Institute of Social Medicine, Occupational Health and Public Health, Medical Faculty, University of Leipzig, Leipzig, Germany

**Keywords:** Public stigma, Social stigma, Long-term care, Informal care, Aged, Gender, Employment

## Abstract

**Background:**

Stigma and informal caregiving are determinants for health and wellbeing, but few studies have examined stigma towards informal caregiving. Public stigma may be expressed differently towards caregivers depending on their gender and employment status due to societal norms. Therefore, this study analyzes if there is a difference in public stigma shown by the general population toward informal caregivers of care recipients aged 65 years or older based on the observed caregiver’s gender or working status.

**Methods:**

A cross-sectional study was conducted in Germany. Data from 1038 adult participants from the general population in Germany were assessed with an Online-Survey. They were recruited with a quota-system based on the German micro census. Participants were randomly assigned to one of 16 vignettes describing a caregiving situation, which varied in the caregiver’s gender and working status, and care recipient’s gender and type of impairment. After reading the vignette, they were asked to provide sociodemographic information and complete three questionnaires on public stigma assessing their emotional (Emotional Reactions), behavioral (Social Distance) and cognitive reaction (Statements on informal caregivers) to the caregiver described in the vignette. Regression analyses, adjusted for sociodemographic data of the participants, were conducted.

**Results:**

Findings indicated an association between reading about male caregivers and increased social distance, compared with reading about female caregivers. Reading about working caregivers was associated with decreased social distance and increased appreciative statements, compared to reading about non-working caregivers. Analyses after stratifying by gender of the caregiver in the vignette indicated an association between reading about female working caregivers and increased appreciative statements, compared to reading about female non-working caregivers. When stratifying by working status, an association was found between reading about male working caregivers and increased social distance, when compared to reading about female working caregivers.

**Conclusions:**

This study’s findings indicate that gender and working status of the perceived informal caregivers are of relevance to the public stigma directed towards these caregivers. Male and non-working informal caregivers were shown more public stigma than female and working informal caregivers. Thus, interventions to reduce public stigma, in particular towards male and non-working caregivers, are recommended.

## Introduction

Due to the ongoing demographic ageing occurring in populations around the globe, the demand for care is increasing [1, 2]. Informal care in particular is expected to gain more relevance, due to the preference that has been shown among the general population for receiving care at home and by relatives [3]. In this study informal care is defined as family care for a person aged ≥65 years (*aged care recipient*), provided by adult children. Adult children are one of the primary resources of informal caregiving [4, 5].

As illustrated in Pearlin’s stress-process model [6], caregiving can be a stressful experience for the caregiver that can result in diminished health and well-being [7, 8]. According to the model, various factors regarding the caregiver, the care recipient and their social context can influence this stress process. Thus, the social evaluation of caregiving by the public could be of relevance in this context as well. A negative social evaluation, in other words, a stigma expressed towards informal caregivers, could contribute to the negative consequences for health and wellbeing that previous research on consequences of informal caregiving has found [7, 8]. However, while various studies have investigated social and health consequences of informal care for caregivers [9, 10] and care recipients [11], very few studies have explored the *stigma* of informal caregiving. Also, there is a lack of studies that explore the evaluation of informal caregivers *by society*, i.e. public stigma. In the following, stigma will be defined and afterwards the previous findings on stigma towards informal caregivers will be reported.

### Definition of stigma

*Stigma* is defined as a negatively perceived attribution of an individual, which changes perceptions of and behavior towards the stigmatized individuals [12]. Based on stigma theory from Goffman, Link and colleagues [12–14], a negative social evaluation of an attribute or behavior is the basis of stigma. The attribute or behavior is associated with specific stereotypes, and results in emotional and behavioral reactions, such as distancing oneself from the stigmatized individual (social distance, e.g., not talking to, not staying in the same room, not befriending the stigmatized person). Previous research found, for example, reports of disgust as emotional reactions, individuals avoiding caregivers and voicing stereotypes such as perceiving caregivers as being neglectful [15]. Thus, stigma can be expressed and assessed by emotional, cognitive, and behavioral responses towards the stigmatized group.

Stigma can be assessed in terms of self-perception of the stigma by the stigmatized individual or in terms of stigma expressed by society towards the stigmatized group. The later refers to *public stigma* [16]. This study focuses on public stigma because it allows the assessment of the social evaluation by the society that is the basis of stigma and can influence other forms of stigma, like self-perceived stigma [12, 16, 17].

### Previous research on stigma in the caregiving context

Previous research has shown that the performance of informal caregiving can have negative psychological consequences [10, 18]. Adding to this, previous research indicates *stigma* towards informal caregiving can have negative consequences for the caregiver’s psychological health and social integration as well [19–21]. Moreover, further findings in different research fields indicate that stigma can inhibit help-seeking behavior in caregivers [22–24]. In addition to the negative consequences of caregiver stigma found for caregivers, negative consequences for care recipients have been indicated as well. For example, Weisman de Mamani and colleagues [25] found that dementia caregivers criticized their care recipients more and reported more emotional overinvolvement with increasing caregiver stigma. The authors assume this to be the result of caregivers trying to control their care recipient’s dementia-related behavior which they perceive as stigmatizing. Moreover, studies found that shame and fear of being judged resulted in caregivers isolating themselves and their care recipients from social contacts [19] and higher caregiver stigma was associated with lower community reintegration of the care recipient [20]. Thus, stigma towards informal caregivers can have detrimental consequences for caregivers and for their care recipients as well. To be able to prevent this, a better understanding of caregiver stigma and factors that may influence this stigma is needed.

Previous research has only focused on self-perceived stigma (e.g., [15, 20, 21, 26]). However, self-perceived stigma does not necessarily correspond with the public stigma. Public stigma towards informal care (*public caregiver stigma*) has not been investigated yet, except by previous research from this project that indicates that public stigma towards informal caregivers occurs [27]. However, knowledge on public stigma is essential, since stigma is by definition based on a negative attribution from society and public stigma can influence other forms of stigma, like self-perceived stigma [12, 16, 17]. Moreover, some previous studies focused only on *courtesy stigma* [15, 19]. This is defined as stigma towards the care recipient that is projected on the caregiver [12, 28]. Thus, it differs from the stigma shown directly towards the caregiver (public stigma).

### Possible determinants of stigma

As Pearlin and colleagues [6] illustrated, informal caregiving is a heterogeneous context, in which characteristics of the caregiver can vary, such as their gender or their employment status. These different characteristics of the caregiver could be influential factors for public stigma. That is, the society may express public stigma towards informal caregivers differently depending on their gender and their involvement in working life. In consequence, some groups of informal caregivers may be more vulnerable towards public stigma and its possible negative consequences. However, there are only a few studies that explore stigma towards informal caregivers, and these differ decidedly in the type of stigma and the perspective they analyzed. Therefore, there is also a lack of studies on possible influential factors on *public caregiver stigma*.

Results from a previous study indicate that female caregivers report more perceived courtesy stigma [21], which supports the assumption that the caregiver’s gender may be of importance for public stigma as well. Gender has been shown to play a relevant role in the informal caregiving context. Traditional gender role expectations place women in the role of the main caregiver [29–31]. This is mirrored in the distribution of caregiving, with the majority of caregivers being women, who provide care more frequently and for longer periods of time than men [32–34]. These different gender-roles-based expectations towards male and female caregivers [29–31] may also influence the societal evaluation, i.e. the public stigma towards these two groups. For example, male caregivers may be seen as acting outside their traditional gender role. Therefore they may be shown higher public stigma than female caregivers, who would be acting within their traditional gender role. Yet, female *working* caregivers may be shown more public stigma than female *non-working* caregivers, since they are acting outside their traditional gender role, i.e. being primarily or solely a caregiver.

Furthermore, there is evidence for differences in the consequences of informal care on male and female caregivers, with women reporting more psychological distress and mental health problems [31, 32, 35, 36]. Different public stigma reactions to both caregiving groups could be a factor that leads to differences in experienced health consequences. Thus, research is needed on possible differences in public caregiver stigma towards male and female caregivers. This could help, first, to gain a better understanding of society’s perspective on informal caregiving by male or female caregivers, and secondly, to provide a basis to develop interventions that approach both groups in accordance with their needs.

We presume the caregiver’s working status may be another important influential factor for public caregiver stigma. The German society is characterized by the belief in a work-centered meritocracy [37], thus, being (an active and productive) part of the work force is essential for one’s social status. This societal perspective may influence the societal evaluation of caregivers who are working or not working, with a higher public stigma to be expected towards non-working caregivers. According to previous research, the majority of informal caregivers are working while caring, and this group has been increasing in size [34]. However, combining caregiving with work has been associated with negative consequences for caregivers’ health compared to non-working caregivers [38–40]. This is in line with the majority of studies showing a decrease in labor market participation [41] or an increasing likelihood for retirement [42] when providing informal care. Increased public stigma towards non-working informal caregivers, for example, could thus cause caregivers to continuing working, despite experiencing burden and negative health consequences. It may also prevent them from using short-term support options based on the *care leave act* (Pflegezeitgesetz) or the *family care leave act* (Familienpflegezeitgesetz). However, some research has also indicated that the combination of work and caregiving can be positive for caregivers, for example, with work being seen as a respite from care [43–46]. Research on possible different public stigma reactions towards working and non-working caregivers may help to gain a better understanding of the society’s view on the combination of informal care and working, and show if one group may be targeted more. These findings can help to develop appropriate interventions to reduce public caregiver stigma. So far, no research regarding stigmatizing reactions towards working or non-working caregivers exists yet.

Thus, this study is an explorative investigation that aims to analyze if the society expresses a different stigma towards female or male, and towards working or non-working caregivers. In other words, this study analyzes if the gender and working status of informal caregivers is of relevance for the public stigma expressed towards them by the general population. Therefore, the association between the caregiver characteristics of gender and working status with the public stigma shown towards informal caregivers of aged care recipients will be analyzed.

## Materials and methods

### Study design and sample

Recruitment and data assessment were conducted by USUMA in cooperation with Lightspeed GMI. Participants of this cross-sectional study were drawn from an Online-Panel, recruited, for example, via social media networks. A quota-system was used, that is, participants were drawn proportionally to the quotas of age, gender and regional distribution of the German population from the German micro census data of 2016. Inclusion criteria were being 18 years or older and living in Germany with online access, i.e. individuals from the general population were drawn as participants. A sample size of 1000 participants was intended, to allow for at least 50 individuals per vignette group (16 vignettes). This was achieved, with the Online-Survey being completed by 1038 participants. The final sample distribution was quite representative for the regional distribution of the adult population living in Germany. Men were slightly overrepresented (104% quota-fulfillment) and women slightly underrepresented (96% quota-fulfillment). Those of younger age (18 to 34 years) were underrepresented - men in particular (quota-fulfillment between 48 and 59%). For those of higher age (75 years and older), men were overrepresented (123% quota-fulfillment) and women underrepresented (35% quota-fulfillment). The Online-Survey was conducted with a computer assisted web interview (CAWI, approximate duration 10 min). All participants gave informed consent. An ethics vote was declared to not be necessary by the ethics committee of the medical chamber of Hamburg (Ärztekammer Hamburg). Further details can be found in the descriptive study [27]. The data are available from the authors upon reasonable request.

### Procedure

Participants of the general population were randomly assigned to one of 16 vignettes. The vignette described a typical situation of informal long-term caregiving for an aged care recipient. To assess the influence of characteristics of the caregiver and the care recipient, the vignette varied across four dimensions: gender (female/male) and working or employment status *of the caregiver* (working or non-working), and gender (female/male) and type of impairment (physical or mental impairment) *of the care recipient*. An example of a vignette is given in Table 1.

**Table 1:** An example of the 16 vignettes describing a typical care situation and being varied regarding caregiver’s gender and working status, as well as care recipient’s gender and type of impairment (German and English version)

Variables	German Version	English Version
*Female, working caregiver; female, physically impaired care recipient*	Sabine pflegt ihre Mutter (70 Jahre). Die verwitwete Mutter hat seit über einem halben Jahr körperliche Schwierigkeiten. Ihre rechte Körperhälfte ist immer mehr in der Beweglichkeit eingeschränkt. Sie kann nur noch mit Unterstützung aufstehen und laufen, und hat nur noch wenig Kraft in der rechten Hand (Rechtshänder). Sabine hat sie nun zu sich ins Haus geholt. Bevor Sabine zur Arbeit geht, bereitet sie Frühstück und Mittagessen zu und hilft ihrer Mutter beim Essen. In der Mittagspause ruft sie zu Hause an, um sich zu erkundigen, ob alles in Ordnung ist. Wenn Sabine zu Hause ist, unterstützt sie sie auch beim Toilettengang, da dieser für die Mutter alleine sehr anstrengend ist. Morgens und abends hilft Sabine ihr beim An- und Ausziehen. Auch beim Waschen des ganzen Körpers hilft sie ihr. Alle zehn Tage nimmt sich Sabine einen halben Tag frei, um ihre Mutter zum Arzt oder anderen Terminen zu bringen. Da die Mutter wenig unternehmen kann, verbringt Sabine jedes Wochenende mit ihr, um ihr Gesellschaft zu leisten.	Sabine cares for her mother (70 years old). Her widowed mother has had physical difficulties for over half a year. The right side of her body is increasingly limited in its mobility. She can only stand up and walk with support and has little strength in her right hand (she is right-handed). Sabine has moved her mother into her home. Before Sabine goes to work, she prepares breakfast and lunch, and helps her mother to eat. In her lunchbreak, she rings home to check that everything is alright. When Sabine is at home, she supports her mother to go to the toilet, as this is very tiring for her mother to do alone. In the morning and evening, Sabine helps her mother to dress and undress. She also assists her mother with full-body washing. Every 10 days, Sabine takes half a day off, to take her mother to the doctor or some other appointments. As her mother cannot do very much, Sabine spends every weekend with her mother, to keep her company.

First, they were provided with a vignette describing the caregiver situation of a specific caregiver, for example, a working male caregiver. After reading the vignette, participants were asked to complete the questionnaire which assessed their public stigma towards the informal caregiver whom they had read about in the vignette.

### Instruments

#### Independent variables

Two dimensions, on which the vignettes varied, were analyzed as independent variables – *caregiver’s gender* and *working status*. The vignettes refer to intergenerational caregiving by adult children for their parents aged 65 years or older. Thus, the caregivers described in the vignette which are female refer to daughters, and those which are male refer to sons of the care recipients. The caregivers described in the vignettes were either working or have stopped working due to caregiving (non-working).

#### Dependent variables

##### Public stigma

*Emotional Reactions to informal caregiving*. Emotional reactions towards informal long-term caregivers of aged care recipients were assessed with an instrument [27] based on the Emotional Reaction to Mental Illness Scale [47]. The instrument consisted of a list of emotions which were assessed in three subscales: devaluing feeling (anger, disgust, envy, guilt, shame, contempt, incomprehension, embarrassment; Cronbach’s α = .80), appreciative feelings (sympathy, desire to help, admiration, happiness, pride, enthusiasm; Cronbach’s α = .80), and regretful and anxious feelings (fear, pity, sadness; Cronbach’s α = .62) [27]. Participants were asked to rate these emotions (Range: 1–5) in regard to the informal caregiver described in the vignette; that is, they were asked to rate their own emotions towards the caregiver described in the vignette. Higher scores indicate higher agreement with the emotions, i.e. feeling these emotions more towards the observed caregiver.

*Behavioral reactions to informal caregiving*. To assess the behavioral reaction indicating a public caregiver stigma, the German translation [48] of the social distance scale [49, 50] was used. The scale included items such as “Would you be comfortable having such a person as a neighbor?”. We added two items to the scale (“Would you enter a romantic relationship with such a person?”; “Would you befriend such a person?”). Items were rated in regard to the informal caregiver from the vignette (9 items, Range:1–5). Thus, the instrument assessed the level of social distance a person would show towards the described caregiver in the vignette. Higher scores indicate higher social distance. The scale has been shown to have good validity and reliability [14]. Our scale showed a good internal consistency of .93 [27].

*Cognitive reaction to informal caregiving.* To assess the cognitive aspect of public caregiver stigma, a list of statements towards informal care for aged care recipients was used. These items were rated in regard to the informal caregiver from the vignette (13 items, Range: 1–5). The instrument was developed and tested for its dimensionality in a former study [27]. The statements were assessed on three subscales: accusing statements (Cronbach’s α = .71) (e.g., “Caregivers provide care because they want to feel needed.”), devaluing statements (Cronbach’s α = .66) (e.g., “Caregivers put themselves into a position where they become a victim.”), and appreciative statements (Cronbach’s α = .66) (e.g., “Caregivers provide a valuable service to society.”). Higher scores indicate higher agreement with the statements.

#### Covariates

##### Sociodemographic data

The participants were asked to give information on age, gender, marital status (married, living together; married, living separately; divorced; widowed; single), and highest educational degree (upper secondary school; qualification for applied upper secondary school; polytechnic secondary school; intermediate secondary school; lower secondary school; currently in school training; without school-leaving qualification).

### Data analysis

The analysis of the public stigma outcomes indicated various outliers. To prevent biased results due to these outliers we winsorized our data by taking the highest or lowest 5% of the data and replacing it with the highest or lowest score within the 95% of the remaining data, respectively.

To test our hypotheses, multiple linear ordinary least square regressions were implemented, which were adjusted for sociodemographic data of the participants. Moderator analyses and stratified analyses were conducted to test if different combinations of the caregiver’s characteristics were associated with stigma towards informal caregiving for older individuals. This allowed us to analyze if public stigma also differs between subgroups in which both characteristics (gender, employment) were taken into account, for example, female working caregivers compared to male working caregivers. To prevent heteroscedasticity, robust standard errors were calculated for all analyses. Partial eta-squared (η^2^) is given for significant regression coefficients.

One person reported ‘diverse’ as a gender category. Since this sample was too small for a subgroup analysis, we excluded this person from our calculations, reducing the analytical sample to 1037 participants. In general, the variables had very few missing values (0.10 to 0.69% missing values). Listwise deletion was employed for observations with missing values. All analyses were conducted with Stata 16.0 (Stata Corp., College Station Texas) and the level of significance was set at α = .05. Hypothesis tests were two-tailed.

## Results

### Descriptive statistics

The results of the descriptive statistics can be found in Table 2. In total, 51.01% (*N* = 529) of the participants were assigned to vignettes describing a female caregiver and 48.99% (*N* = 508) to vignettes describing a male caregiver, and 49.76% (*N* = 516) were assigned to the vignette describing a working caregiver and 50.24% (*N* = 521) to vignettes describing a non-working caregiver. The participants were on average 52.33 (SD = 16.61) years old and about half of them were female (48.99%). In total 60.46% were married and living with their partner, with 21.22% being single. Currently employed were 53.23%, while 32.40% were already retired and 14.37% were not employed.

**Table 2:** Descriptive statistics of the complete sample (M = Mean; SD = standard deviation)

***Stigma***	***M(SD) / N(%)***
Emotional reaction to informal caregiving	
- Devaluing feelings	1.39 (.45)
- Appreciative feelings	3.60 (.78)
- Regretful and anxious feelings	2.68 (.93)
Social distance	1.89 (.69)
Cognitive reaction to informal caregiving	
- Accusing statements	2.03 (.63)
- Devaluing statements	2.20 (.71)
- Appreciative statements	4.34 (.63)
***Sociodemographic data***	
N	1037
Age	52.33 (16.61)
Gender (female)	508 (48.99)
Education – highest educational degree	
- Upper secondary school	360 (34.72)
- Qualification for applied upper secondary school	115 (11.09)
- Polytechnic Secondary School	80 (7.71)
- Intermediate Secondary School	331 (31.92)
- Lower Secondary School	144 (13.89)
- Currently in school training/education	4 (0.39)
- Without school-leaving qualification	2 (0.19)
Employment status	
- Employed	552 (53.23)
- Retired	336 (32.40)
- Not employed	149 (14.37)
Marital status	
- Married, living together	627 (60.46)
- Married, living separately	41 (3.95)
- Divorced	102 (9.84)
- Widowed	47 (4.53)
- Single	220 (21.22)
***Vignettes***	
Gender of the caregiver	
- Female	529 (51.01)
- Male	508 (48.99)
Working status of the caregiver	
- Yes, working	516 (49.76)
- No, non-working	521 (50.24)
Gender of the care recipient	
- Female (mother)	529 (51.01)
- Male (father)	508 (48.99)
Type of impairment of the care recipient	
- Physical impairment	546 (52.65)
- Mental impairment	491 (47.35)

### Multiple regression analyses

Results of the adjusted multiple regression analyses can be found in Table 3. Findings indicate that reading about a male caregiver, compared to reading about a female caregiver, was significantly associated with increased *social distance* scores (b = .13, *p* = .004, CI[.04; .21], η^2^ = .008). Furthermore, reading about a working caregiver, compared to reading about a non-working caregiver, was significantly associated with decreased *social distance* scores (b = −.11, *p* = .011; CI[−.19; −.02], η^2^ = .006). Reading about individuals who were working while providing care was significantly associated (b = .12, *p* = .002, CI[.04; .19], η^2^ = .009) with increased *appreciative statements*.

**Table 3:** Results of the multiple regression analyses with emotional, behavioral, and cognitive public caregiver stigma as outcomes and gender and working status of the caregivers as main independent variables, adjusted for the sociodemographic background of the participants

Outcome variables	Devaluing feelings	Appreciative feelings	Regretful and anxious feelings	Social distance	Accusing statements	Devaluing statements	Appreciative statements
Model	(1)	(2)	(3)	(4)	(5)	(6)	(7)
Caregiver’s gender (Ref. female)	0.02	−0.02	−0.00	0.13**	0.04	0.01	−0.05
	(0.03)	(0.05)	(0.06)	(0.04)	(0.04)	(0.04)	(0.04)
Caregiver’s working status (Ref. no, non-working)	−0.04	0.09+	0.07	−0.11*	−0.02	− 0.00	0.12**
	(0.03)	(0.05)	(0.06)	(0.04)	(0.04)	(0.04)	(0.04)
Constant	1.74***	3.20***	3.14***	2.07***	1.96***	2.14***	4.05***
	(0.07)	(0.12)	(0.14)	(0.11)	(0.10)	(0.11)	(0.10)
Observations	1028	1027	1031	1033	1027	1032	1033
R^2^	0.053	0.050	0.050	0.025	0.011	0.028	0.056

Unstandardized regression coefficients and robust standard errors in parentheses. Emotional reactions to informal caregiving (devaluing feelings, appreciative feelings, regretful and anxious feelings), Range 1–5, higher scores indicating higher agreement with the emotions; behavioral reactions to informal caregiving (social distance), Range 1–5, higher scores indicating higher social distance; cognitive reactions to informal caregiving (accusing statements, devaluing statements, appreciative statements), Range 1–5, higher scores indicating higher agreement with the statements. Sociodemographic information on the participants was included as covariates (age, gender, education, and marital status). Level of significance: *** *p* < 0.001, ** *p* < 0.01, * *p* < 0.05, + *p* < 0.10

### Stratified and moderator analyses

In Table 4 the results of the analyses stratified by working status of the described caregivers are given. Results of the analyses stratified by gender can be found in Table 5.

**Table 4:** Results of the multiple regression analyses with emotional, behavioral, and cognitive public caregiver stigma as outcomes, stratified by caregiver’s working status, with caregiver’s gender as main independent variables and adjusted for the sociodemographic background of the participants

Outcome variables	Devaluing feelings		Appreciative feelings		Regretful and anxious feelings		Social distance		Accusing statements		Devaluing statements		Appreciative statements	
	working	non-working	working	non-working	working	non-working	working	non-working	working	non-working	working	non-working	working	non-working
Model	(1)	(2)	(3)	(4)	(5)	(6)	(7)	(8)	(9)	(10)	(11)	(12)	(13)	(14)
Caregiver’s gender (Ref. female)	0.02	0.01	−0.04	−0.01	− 0.02	0.00	0.14*	0.11+	0.04	0.05	0.02	0.00	−0.10+	−0.01
	(0.04)	(0.04)	(0.07)	(0.07)	(0.08)	(0.08)	(0.06)	(0.06)	(0.06)	(0.06)	(0.06)	(0.06)	(0.05)	(0.06)
Constant	1.63***	1.81***	3.19***	3.30***	2.94***	3.41***	1.97***	2.08***	1.83***	2.07***	1.95***	2.32***	4.12***	4.11***
	(0.10)	(0.10)	(0.17)	(0.16)	(0.20)	(0.20)	(0.15)	(0.16)	(0.14)	(0.15)	(0.16)	(0.16)	(0.13)	(0.14)
Observations	515	513	512	515	514	517	516	517	511	516	516	516	516	517
R^2^	0.071	0.076	0.048	0.076	0.058	0.068	0.037	0.022	0.027	0.016	0.026	0.054	0.055	0.066

Unstandardized regression coefficients and robust standard errors in parentheses. Emotional reactions to informal caregiving (devaluing feelings, appreciative feelings, regretful and anxious feelings) Range 1–5, higher scores indicating higher agreement with the emotions; behavioral reactions to informal caregiving (social distance) Range 1–5, higher scores indicating higher social distance; cognitive reactions to informal caregiving (accusing statements, devaluing statements, appreciative statements) Range 1–5, higher scores indicating higher agreement with the statements. Sociodemographic information on the participants was included as covariates (age, gender, education, and marital status). Level of significance: *** *p* < 0.001, ** *p* < 0.01, * *p* < 0.05, + *p* < 0.10

**Table 5:** Results of the multiple regression analyses with emotional, behavioral and cognitive public caregiver stigma as outcomes, stratified by caregiver’s gender, with caregiver’s working status as main independent variables and adjusted for the sociodemographic background of the participants

Outcome variables	Devaluing feelings		Appreciative feelings		Regretful and anxious feelings		Social distance		Accusing statements		Devaluing statements		Appreciative statements	
	female	male	female	male	female	male	female	male	female	male	female	male	female	male
Models	(1)	(2)	(3)	(4)	(5)	(6)	(7)	(8)	(9)	(10)	(11)	(12)	(13)	(14)
Caregiver’s working status (Ref. no, non-working)	−0.04	−0.05	0.09	0.10	0.08	0.04	−0.11+	−0.10+	−0.02	−0.02	−0.00	0.00	0.14**	0.07
	(0.04)	(0.04)	(0.07)	(0.07)	(0.08)	(0.08)	(0.06)	(0.06)	(0.06)	(0.06)	(0.06)	(0.06)	(0.05)	(0.06)
Constant	1.93***	1.58***	3.11***	3.29***	3.22***	3.07***	2.18***	2.08***	2.07***	1.88***	2.31***	1.95***	3.97***	4.11***
	(0.10)	(0.10)	(0.16)	(0.17)	(0.19)	(0.22)	(0.14)	(0.16)	(0.14)	(0.15)	(0.15)	(0.16)	(0.14)	(0.14)
Observations	525	503	522	505	526	505	527	506	524	503	527	505	528	505
R^2^	0.095	0.034	0.091	0.042	0.067	0.061	0.028	0.023	0.012	0.020	0.045	0.039	0.083	0.068

Unstandardized regression coefficients and robust standard errors in parentheses. Emotional reactions to informal caregiving (devaluing feelings, appreciative feelings, regretful and anxious feelings) Range 1–5, higher scores indicating higher agreement with the emotions; behavioral reactions to informal caregiving (social distance) Range 1–5, higher scores indicating higher social distance; cognitive reactions to informal caregiving (accusing statements, devaluing statements, appreciative statements) Range 1–5, higher scores indicating higher agreement with the statements. Sociodemographic information on the participants was included as covariates (age, gender, education, and marital status). Level of significance: *** *p <* 0.001, ** *p <* 0.01, * *p <* 0.05, + *p <* 0.10

When stratifying by working status, reading about male working caregivers was associated with increased *social distance* (b = .14, *p* = .019; CI[.02; .26], η^2^ = .011) compared to reading about working female caregivers. No significant association was found between gender and *social distance* in the non-working subgroup (b = .11, *p* = .084) (Table 4).

When stratifying by gender (Table 5), reading about working female caregivers was significantly associated with increased *appreciative statements* (b = .14, *p* = .007; CI[.04; .25], η^2^ = .014), compared to reading about non-working female caregivers.

No significant interaction was found between gender and working status for the emotional reaction outcomes *devaluing feelings* (b = −.01, *p* = .817), *appreciative feelings* (b = .00, *p* = 1.00), or *regretful and anxious feelings* (b = −.04, *p* = .706). For the outcome *social distance* no significant interaction was found between gender and working status of the caregiver (b = .01, *p* = .897). No significant interaction effect between gender and working was found for the outcome *accusing statements* (b = .00, *p* = .968), *devaluing statements* (b = .01, *p* = .948) or *appreciative statements* (b = −.07, *p* = .382).

## Discussion

### Summary

This is the first study investigating the association of the informal caregiver’s characteristics of gender and working status with public stigma shown towards caregivers providing care for aged care recipients. Results point to the relevance of these characteristics for behavioral and cognitive public caregiver stigma. Male caregivers were shown more social distance, compared to female caregivers. Caregivers who were working while caring, compared to those non-working, were shown less social distance and more appreciative statements. Although no significant moderation by any of the caregiver’s characteristics was found, results from stratification suggest that among working caregivers, men are shown more stigma than woman, and among female caregivers those who are working while caring, are shown less stigma than those who were not working while caring. The results will be discussed in detail in the following sections.

### Discussion of the association between the caregiver’s gender and public stigma

Interestingly, former research indicated that women *perceived* more courtesy stigma towards themselves when caring for dementia care recipients [21]. Yet, our results indicate that men are *shown* more stigmatizing reactions from society than women. Thus, our results point towards the difference that can exist between varying types of stigma and emphasize the importance of analyzing stigma from different perspectives. Further research is recommended to analyze if male informal caregivers may also perceive that more public stigma is directed towards them than towards women. One study indicates that male caregivers perceive more affiliate stigma (affiliate stigma represents the internalized negative public perceptions towards caregivers), which supports this assumption [51].

A possible explanation for increased public stigma towards male compared to female caregivers may be expectations of gender-role-conforming behavior. Men who provide care may be seen as acting outside their traditional role as provider and taking on women’s traditional role of caregiver [29–31]. This is supported by previous research indicating that men reinterpret caregiving tasks in the context of traditional male gender roles to be able to accept the caregiving role [52]. For example, they perceive themselves as the only one capable of providing care because it requires the physical strength of a man, or they perceive caregiving as an extension of their responsibility as a man and husband to look after and provide for their wife. Female caregivers act in accordance with their traditional gender role and may therefore be shown less stigma than men.

In light of the current caregiving situation these different public stigma reactions to male and female caregivers are problematic. Previous research indicates that men already struggle with caregiving due to its incongruence with their traditional gender role as well as with practical difficulties in performing care tasks [52–55]. Stigmatization of male informal caregivers may thus further impede men from becoming involved in caregiving if other potential caregivers are available. For men who have no choice but to become a caregiver, being stigmatized for caregiving may result in more social isolation and worse mental health, as has been found in research on stigma before [20, 21]. Thus, stigma may be a risk factor that could further worsen negative consequences for caregiver’s health and wellbeing which often result from caregiving performance [7, 8, 10, 18]. However, this was not analyzed in this study, thus, further research in this regard is recommended. Moreover, stigma can deter individuals from seeking help [22–24]. Male informal caregivers may therefore be more hesitant to ask for help with caregiving tasks or support in coping with the caregiving situation in general, such as using counseling services, as has been found previously [56, 57]. Being shown more social distance than female caregivers, could also be responsible for a decrease in social support. This is problematic for male caregivers in particular, since research indicates that men already have a smaller social network than women [58] and lower social support is associated with increased caregiver burden [59, 60]. Thus, public stigma could lead to increasingly negative consequences for male caregivers.

Moreover, female caregivers may suffer as well from these different public stigma reactions. Currently the majority of informal caregivers are still women [33, 34], and despite men becoming more involved recently [61], being more stigmatized for providing care may act as a barrier for men to take on the caregiver role. This may contribute to a continuation of the current unequal distribution of caregiving between men and women. In light of findings of worse psychological consequences and burden for female caregivers [32, 35, 36], this is a worrisome result. Thus, increased public stigma towards male caregivers may easily lead to detrimental consequences for male as well as female informal caregivers.

### Discussion of the association between the caregiver’s working status and public stigma

Working caregivers were associated with lower stigma in terms of less social distance and more appreciative cognitive reactions than non-working caregivers. This is in line with and may even be an influential factor for the increase of the group of individuals combining work and care in the last decade [34]. The results may therefore indicate a societal preference for the combination of work and caregiving in line with the expectations of a meritocracy, i.e. being a productive part of the work force.

That working caregivers are less stigmatized may indicate that they are less endangered by the possible negative consequences of stigma [20, 21]. Moreover, findings from previous research indicate that being employed while performing informal care can actually be a resource for informal caregivers [43, 44]. Thus, the results indicate that public caregiver stigma is not a barrier to combining working and caregiving. Instead, low public stigma and higher appreciative statements towards working caregivers may indicate societal interest in enabling caregivers the combination of caregiving and work. This could be a good basis for policy changes and interventions to enable and support this.

However, there is also evidence that working caregivers experience higher burden [40] and decreased health compared to non-working caregivers [38, 39]. Moreover, the majority of previous research indicates a decrease of working performance and participation among informal caregivers [41], which has been shown for intensive caregiving in particular [41, 62, 63]. These findings indicate that the combination of care and work can be detrimental for informal caregivers. Higher public stigma towards non-working caregivers may therefore encourage individuals to keep combining work and caregiving, despite the negative consequences of this combination. In consequence, public caregiver stigma could worsen the situation for working caregivers. Additionally, for those caregivers who are not working, the situation may also worsen due to higher public stigma which could increase caregiver burden and impair their health and social integration, as has been found for other forms of stigma [20, 21]. Interventions to reduce public stigma towards non-working informal caregivers are therefore urgently recommended. Possible interventions are described in the section on implications.

### Discussion of the results from stratification and moderator analyses

Previous research indicates that male caregivers are slower to retire, while female caregivers are more likely to retire earlier [64]. This behavior is in line with traditional role distributions and expectations with men as provider and women as caregiver [29, 64]. Thus, one may have expected working women to be shown more public stigma and men less. However, our results indicate the situation to be more complex.

Results from stratification suggest the following: 1) *working female* caregivers were associated with increased appreciative statements, compared to *non-working female* caregivers, and 2) *working male* caregivers were associated with *increased* social distance compared to *working female* caregivers.

Firstly, while no significant interaction effect was found, these results indicate that female caregivers are more appreciated when working than when they are non-working. This is in line with former results of this study indicating working caregivers were in general associated with less social distance and more appreciative statements than non-working caregivers. However, women providing care while continuing to work are not in line with traditional role expectations [29, 30]. There is still a vast array of evidence of discrimination of women in the labor market (e.g. maternal wall [65, 66], glass ceiling [67]), indicating that traditional gender roles and expectations based on them are still present. However, with the increasing participation of women in the labor market, women are taking on more tasks that were traditionally performed by men [30, 61], which at least supports an increasing flexibility in female gender roles. Our findings may thus be the result of an increasing flexibility or even small changes in gender roles by showing that working female caregivers may not only be accepted but seemingly even more appreciated than non-working female caregivers. However, as explained before, the combination of work and caregiving can have detrimental health effects for caregivers [38–40]. Thus, higher appreciation may not necessarily have a mitigating effect on stress and burden of female working caregivers, instead, it may worsen it, for example, by preventing them to implement adaptions to their working life, such as a reduction of working time. In consequence, while appreciation may itself be positive, it can still have a negative effect on caregivers.

Secondly, while no significant interaction effect was found, the results still indicate a more negative reaction towards male working caregivers, compared to female working caregivers. This supports former results of this study showing more stigma of male caregivers in general. Adding to this, results from stratification suggest that even when men continue working while caregiving, and thereby conform more closely to their traditional role as provider [29, 30], they are still stigmatized. Thus, while women extending their gender roles may be appreciated, men seem to be stigmatized for leaving as well as for extending their traditional gender roles. This is in line with findings showing that men reinterpret their caregiver role in the context of masculine gender identity norms, which indicates an internalized stigma or at least an awareness of stigma towards men performing tasks traditionally performed by women [52]. Further support is given by other research on legal practice in the US showing that men face more discrimination than women when taking on traditional female tasks, such as caregiving for children or sick relatives [68, 69].

Especially in light of previous research showing men to be more likely to continue working when providing care [64], this public stigma reaction is rather problematic. As explained before, this public stigma towards working male caregivers may be a barrier for men to take on caregiving at all and support further gender-role-conform behavior of men, which can have negative consequences for all informal caregivers.

In sum, the results can be seen as indicating different trends regarding gender roles which are expressed in different public caregiver stigma reactions towards male and female (working) caregivers. More research on this is recommended.

### Limitations and recommendations for further research

The study has a few limitations. A convenience sample was used, restricting the representativeness and thus the generalizability of the results. However, the sample was drawn with a quota-system based on the German micro census data of 2016 and is still quite representative of this population. Furthermore, due to being assessed online, the social de-contextualization effect that have been found for Online-Surveys may have helped to decrease or even prevent a social desirability effect [70].

The effect sizes that were found in this study are small. Furthermore, being a cross-sectional study, drawing causal conclusions is difficult and should be considered with caution. Further research with a longitudinal design is needed. However, it should be noted that in light of the general lack of research in this area this exploratory study is still the first to provide findings regarding the association between *caregiver characteristics* and the *public stigma* towards caregivers of *aged care recipients*.

Also, no significant interaction effects were found. This may be traced back to a power problem, with the sample being too small to find these effects. Therefore, results from stratified analyses only indicate tendencies and have to be interpreted cautiously, and further research with a larger sample is recommended.

Last, further research is recommended which takes other aspects of the caregiving situation into account regarding their potential relevance for public stigma towards informal caregiving. Thus, further research is needed which varies other caregiver characteristics, such as the caregivers socioeconomic background (e.g., income) and the type of care relationships in the vignettes. Since we had to limit the number of vignette variations, in order to still be able to test different combinations of variations with an adequate sample size, this study did not include further variations of caregiver characteristics. Also, the study focused only on adult child caregivers. They are among those most often involved in informal caregiving [4, 5] and being on average younger than partner caregivers [5], it is expected that they may be confronted more and for a longer time with a combination of caregiving and employment [34] and a potential public stigma towards this form of caregiving. Thus, the public stigma towards this group of caregivers is of particular interest.

## Conclusion and implications for research and practice

This study is the first to provide evidence regarding the association between the caregiver’s gender and working status and public stigma shown towards the informal caregiver of individuals aged 65 years and older. The results indicate that gender as well as working status of the caregiver are associated with public caregiver stigma. Increased public stigma towards male caregivers compared with female caregivers was shown. Also, lower public stigma and higher appreciation towards working caregivers compared to non-working caregivers was found. Results from stratification add to this by indicating that even male working caregivers are shown more public stigma, and female caregivers that are working (compared to female non-working caregivers) are shown more appreciation.

These results provide insights into a new field of research. They indicate that public stigma shown by society can differ from stigma as perceived by the stigmatized individual, and that characteristics of the stigmatized caregivers play a relevant role. Self-perceived caregiver stigma has been shown to result in negative health and social consequences [20, 21], but self-perceived stigma is influenced by public stigma. Thus, this study provides further information on the basis of self-perceived stigma and on factors that can influence stigma. It can thereby help to point out which groups may be targeted specifically and may thus be more vulnerable to perceive caregiver’s stigma and subsequently experience negative health and social consequences. Therefore, our findings indicate the need for further research in this field and on different forms of stigma to extend our current understanding of caregiver stigma and possible influential factors. This study indicates that the caregiver’s gender and working status seem to be relevant factors regarding the public stigma expressed by society. As discussed, society’s understanding of male and female gender roles may be responsible for this. Further research regarding gender roles in this context is needed to gain a better understanding of the relevant aspects for public caregiver stigma. Furthermore, while these results may be representative of other cultures with similar norms and societal expectations, further research regarding these associations in different cultures is recommended.

Regarding practical implications, interventions to reduce public stigma towards informal caregivers should be aimed primarily at the general population, though some suggestions are also made for interventions that target caregivers themselves. The results suggest a need for interventions to reduce public stigma among the general population directed towards male caregivers. Social marketing campaigns aimed at increasing acceptance of male caregivers, for example, by raising awareness of men that already perform care, could help to achieve this. Furthermore, an intervention aimed at caregivers is recommended, in terms of supporting the more equal distribution of care tasks among female and male caregivers, if both are available, such as son and daughter(-in-law). This could help to demonstrate that caregiving can be performed by both, women and men, and may help men to overcome barriers against taking on caregiving themselves and accepting other male caregivers. For the development of appropriate interventions, further research is recommended on the aforementioned assumptions regarding the role of gender norms and other possible underlying mechanisms of increased public stigma towards male caregivers.

Moreover, the results indicate a societal preference for the combination of work and caregiving. Previous studies support this preference by indicating that caregivers can benefit from continuing to work [43, 44]. Further investigation of these preferences is recommended and could help to inform the development of support options for informal caregivers. *If* this preference is mirrored by caregivers themselves, more effort should be directed into supporting caregivers in combining caregiving and work life.

However, in light of the negative health consequences that have been shown for the combination of work and caregiving [38–40], it is essential to enable caregivers a choice regarding combining work and caregiving that is not biased by public stigma. Thus, public stigma towards non-working caregivers should be targeted with interventions, to prevent possible negative stigma consequences [20, 21] for this group. Moreover, the awareness and understanding of difficulties and negative consequences of informal caregiving (e.g., increased burden and worse health), particularly if it is combined with working, needs to be increased. This could help to broaden society’s understanding of the caregiver’s situation which could thereby help to reduce public stigma directed towards informal caregivers by society.

## Data Availability

The datasets used and/or analysed during the current study are available from the corresponding author on reasonable request.
